# Protein and Organic-Molecular Crystallography With 300kV Electrons on a Direct Electron Detector

**DOI:** 10.3389/fmolb.2020.612226

**Published:** 2021-01-06

**Authors:** Kiyofumi Takaba, Saori Maki-Yonekura, Satoru Inoue, Tatsuo Hasegawa, Koji Yonekura

**Affiliations:** ^1^Biostructural Mechanism Laboratory, RIKEN SPring-8 Center, Sayo, Japan; ^2^Department of Applied Physics, The University of Tokyo, Tokyo, Japan; ^3^Advanced Electron Microscope Development Unit, RIKEN-JEOL Collaboration Center, RIKEN Baton Zone Program, Sayo, Japan; ^4^Institute of Multidisciplinary Research for Advanced Materials, Tohoku University, Sendai, Japan

**Keywords:** DE64, energy filter, electron 3D crystallography (3D ED/MicroED), eEFD, CRYO ARM

## Abstract

Electron 3D crystallography can reveal the atomic structure from undersized crystals of various samples owing to the strong scattering power of electrons. Here, a direct electron detector DE64 was tested for small and thin crystals of protein and an organic molecule using a JEOL CRYO ARM 300 electron microscope. The microscope is equipped with a cold-field emission gun operated at an accelerating voltage of 300 kV, quad condenser lenses for parallel illumination, an in-column energy filter, and a stable rotational goniometer stage. Rotational diffraction data were collected in an unsupervised manner from crystals of a heme-binding enzyme catalase and a representative organic semiconductor material Ph-BTBT-C10. The structures were determined by molecular replacement for catalase and by the direct method for Ph-BTBT-C10. The analyses demonstrate that the system works well for electron 3D crystallography of these molecules with less damaging, a smaller point spread, and less noise than using the conventional scintillator-coupled camera.

## Introduction

Electrons are scattered by light atoms 4–5 orders of magnitude more strongly than X-rays. This property makes electron crystallography applicable to undersized crystals of various samples, which are hard to grow to a suitable size for X-ray diffraction even with a high-intensity synchrotron radiation beam. Thus, this technique known as electron 3D crystallography/3D ED/MicroED is recognized as being more important in synthetic chemistry, material sciences, and related areas, as single particle analysis can be used for larger-sized protein and protein complexes.

For collection of 3D diffraction data from single sample crystal, the crystal has to be rotated, while sequential frames of diffraction patterns are recorded on a detector using an electron microscope (e.g., Nannenga et al., 2014a; Yonekura et al., 2015; van Genderen et al., 2016). The crystal structures can be solved from these electron diffraction patterns as in X-ray crystallography. Like all other cryo-EM techniques, this is based on an assumption that single electron interacts with the sample only once. Rotational and precession techniques could reduce the effect by multiple interactions of single electron known as dynamical scattering in recored patterns (e.g., Vincent and Midgley, 1994; Oleynikov et al., 2007; Nannenga et al., 2014a, 2018), and this effect is not very severe in thin protein crystals composed of light atoms and with high mosaicity (Yonekura et al., 2015). However, the strong interaction of electrons with atoms imposes a limit on the thickness of the sample or the path length of electrons through the sample, and this worsens when the 3D crystal tilts. Indeed, protein 3D crystallography suffer from less incoming electrons to the detector at higher tilt angles. Inelastic scattering is also problematic for thick crystals and/or highly-tilted crystals, due to the shorter mean free path of inelastically scattered electrons compared to elastically scattered ones (Angert et al., 1996). This problem can be eased with use of higher-energy electrons and an energy filter.

For water, 300 kV electrons have ~5.9 and ~1.9 times higher penetrating power than 100 and 200 kV electrons, respectively (ICRU, 2014; Yonekura et al., 2019), and at the same time radiation damage can be reduced with higher-energy electrons (Yonekura et al., 2019). Energy filtration is also powerful particularly for frozen-hydrated crystals. It can effectively remove energy-loss electrons coming largely from amorphous ice surrounding and inside the crystal. This leads to a substantial decrease of the background noise and an improvement of signal-to-noise ratios in diffraction patterns (Yonekura et al., 2002, 2015, 2019; Maki-Yonekura et al., 2020). Moreover, energy filtration removes multiple scattered electrons, as they are likely to be inelastically scattered at least once, while multiple elastically-scattered electrons remain in recorded patterns. Indeed, combination of 300 kV electrons and energy filtering is highly beneficial to achieve higher-quality structure analysis (Yonekura et al., 2015, 2019). We named this approach as eEFD (electron energy-filtered diffraction of 3D crystals) (Yonekura et al., 2019).

The detector technology would be another important element to further improve data quality. Scintillator-coupled CCD (e.g., Yonekura et al., 2015)/CMOS detectors (e.g., Nannenga et al., 2014a), and direct detection detectors (DDD; e.g., van Genderen et al., 2016) have been introduced in the field so far. DDD cameras have high sensitivity for electrons and could be useful for recording weak diffraction spots. There are two major types of DDD cameras, the hybrid-pixel detector, and active pixel sensor. The former includes Medipix/Timepix (Amsterdam Scientific Instruments; X-Spectrum GmbH) and EIGER (DECTRIS Ltd.), which have already been used in many electron diffraction studies done mainly at 200 kV (e.g., van Genderen et al., 2016; Cichocka et al., 2018; Clabbers et al., 2018, 2019; Bücker et al., 2020). These detectors have multiple sensors with a larger pixel spacing (55–75 μm). Each sensor is made of thick silicone crystal, which most incident primary electrons do not penetrate through, yielding a better estimate of deposited electron intensity yet broader spreads among neighboring pixels with higher-energy electrons, 200 and 300 kV (Tinti et al., 2018). Thus, the hybrid-pixel detector would be suitable for lower-energy electrons (≤ 100 kV; Naydenova et al., 2019).

K2/3 (GATAN), Falcon series (Thermofisher scientific), and DE64 (Direct Electron) are widely used in single particle cryo-EM. These detectors are categorized as the active pixel sensor that is made up of a thin layer of silicone crystal and allows incident electrons to pass through. The active-pixel sensor does not seem ideal for electron diffraction due to Landau noise resulting in a relatively poorer estimation of spot intensity, and also the resilience to radiation is thought to be low such that they could not withstand the strong intensity of high energy electrons around a direct beam. One report, however, showed that the Falcon III detector can reduce electron dose needed for protein crystallography with 200 kV electron beam (Hattne et al., 2019).

Here we test another active pixel detector DE64 with a JEOL CRYO ARM 300 electron microscope for electron 3D crystallography. The microscope is equipped with a cold-field emission gun operated at an accelerating voltage of 300 kV, quad condenser lenses for parallel illumination, an in-column energy filter, and a stable rotational goniometer stage. We have already reported the performance of this system for both imaging with a DDD and diffraction with a scintillator coupled CMOS camera (Hamaguchi et al., 2019; Yonekura et al., 2019; Maki-Yonekura et al., 2020). We also developed an unsupervised scheme for data collection of rotational electron diffraction patterns (Takaba et al., 2020). This report presents application of this system with the DE64 detector to crystals of a heme-binding enzyme catalase and a representative organic semiconductor material, 2-decyl-7-phenyl[1]-benzothieno[3,2-*b*][1]benzothiophenes (Ph-BTBT-C10; Minemawari et al., 2014).

## Materials and Methods

### Instrumentation

**Figure 1:**
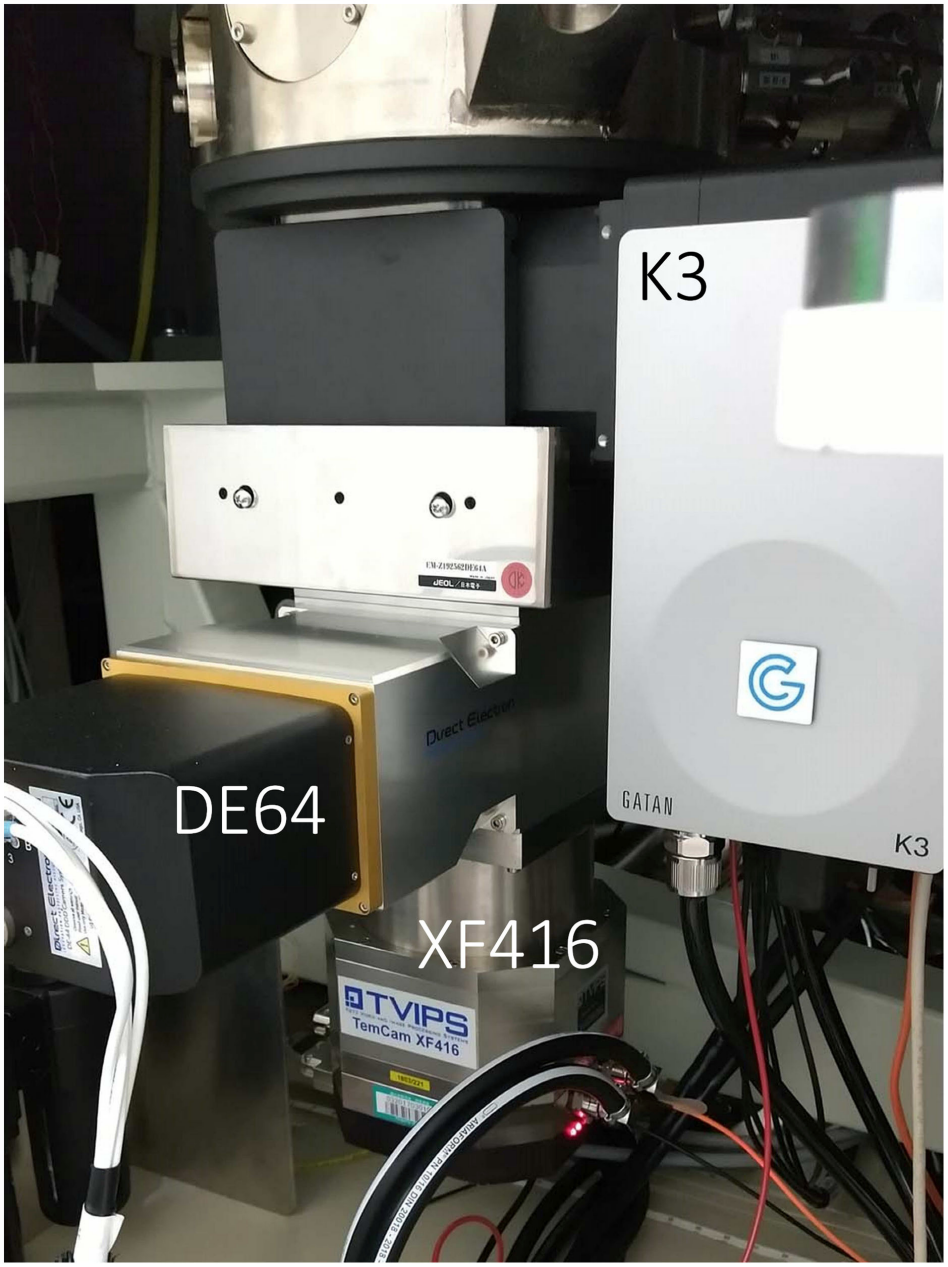
Camera setup in a CRYO ARM 300 electron microscope at RIKEN SPring-8 Center. A GATAN K3 (upper), Direct Electron DE64 (middle), and TVIPS XF416 (lower) were installed below the column of the electron microscope.

A direct detection detector (DDD) DE64 (Direct Electron) was bottom-mounted below a K3 (GATAN) camera under the column of a CRYO ARM 300 microscope (Figure 1). The DE64 has an active pixel sensor of 8k × 8k pixels, and one-pixel size is 6.5 × 6.5 μm^2^. The detector operates in two modes, counting and integration, and the integration mode was used for recording diffraction patterns. A scintillator-coupled CMOS camera, TVIPS XF416 (4k × 4k pixels with a pixel size of 15.5 × 15.5 μm^2^) is also placed under the DE64.

### Sample Preparation

Small thin crystals of catalase were prepared as described previously (Dorset and Parsons, 1975; Yonekura et al., 2015). A few microliters of catalase crystal solution were applied onto a continuous or holey carbon film-coated copper grids with 200 mesh. We usually use Maxtaform HF34 grids, which have a larger mesh size and so is more adaptive to data collection from high-tilt angles. Quantifoil R1.2/1.3 on HF34 grids was suitable for our application. The grid was blotted manually with filter paper and frozen in liquid nitrogen.

Ph-BTBT-C10 powder was synthesized as in (Inoue et al., 2015) and dissolved in chloroform. The solution was directly applied onto a carbon holey film-coated grid and dried. Crystals of Ph-BTBT-C10 were grown on the carbon film.

### Data Collection

Crystals were examined using the JEOL CRYO ARM 300 microscope at a specimen temperature of ~96 K for catalase and at room temperature for Ph-BTBT-C10. SerialEM (Mastronarde, 2005) was used for taking an overview of the entire grid, rough search of crystals, eucentric alignment for grid squares having good-looking crystals, and queuing crystal positions (Takaba et al., 2020). Then, a semi-automated data collection was carried out by combined use of SerialEM and ParallEM (Hamaguchi et al., 2019; Yonekura et al., 2019; Maki-Yonekura et al., 2020), as described (Takaba et al., 2020). Briefly, a sample crystal was sequentially positioned from the queue with SerialEM and rotational frames were continuously collected from each crystal with ParallEM. The diffraction patterns were recorded with a nominal camera length of 3,000 and 500 mm for catalase and Ph-BTBT-C10, respectively, on a hardware-binned 4k × 4k pixel array of the DE64. The detector was operated at 20 frames/s (fps) in integration mode. For catalase crystals, an energy slit was inserted to select only electrons with energy loss <20 eV, which can exclude most electrons with plasmon loss in ice (Langmore and Smith, 1992) and carbon. The energy slit was not used for data collection from Ph-BTBT-C10 crystals in this study, as a field of the view cut was large with this short camera length (Maki-Yonekura et al., 2020). A center beam stopper was always inserted during data collection. The crystal was parallel illuminated with a ~5 μm beam for both the samples and with a selected area aperture of ~1 μm for Ph-BTBT-C10. The goniometer stage was rotated at 2.0°/s for catalase and 1.0°/s for Ph-BTBT-C10 from −68 to 68°. Raw frames were summed and saved as a movie stack, each summed frame of which covered 0.5° rotation. The total dose per summed frame was ~0.005 electrons/Å^2^ for catalase and ~0.01 electrons/Å^2^ for Ph-BTBT-C10, respectively (Tables 1, 2).

**Table 1:** Data collection, crystallographic, and refinement statistics of catalase.

**Sample**		**Catalase (full rotational frames)**	**(first half frames)**
**Data collection**			
Microscope		CRYO ARM 300	
Wavelength (Å)		0.0197	
Electron voltage (kV)		300	
Energy filtration		+	
Camera		DE64	
Camera distance			
	IL1 (hexadecimal)	5524	
	Nominal (mm)	3,000	
	Measured (mm)	4,027	
	Refined (mm)^*^	4,036	
Temperature (K)		~96	
Rotation speed (°/s)		2.0	
Rotation range		−68 to 68°	−68 to 0°
Rotation/frame (°/frame)		0.5	
Dose/frame (e^−^/Å^2^/frame)^†^		0.005	
Total datasets collected		53	
**Crystallographic parameters**			
Space group		*P*2_1_2_1_2_1_	
Cell dimensions			
	*a, b, c* (Å)	70.0, 174.2, 199.5	
	*α, β, γ* (°)	90, 90, 90	
Resolution (Å)		131.3–3.00	131.3–3.00
		(3.17–3.00)^‡^	(3.17–3.00)^‡^
Completeness (%)		82.3 (82.7)	66.5 (66.6)
CC_1/2_ (%)		68.8 (18.5)	73.8 (14.3)
*R*_merge_^§^		1.17 (1.81)	1.04 (1.54)
*I*/σ		3.8 (1.9)	3.0 (1.8)
Multiplicity		43.6 (42.2)	23.2 (22.6)
Solvent content (%)		51.5	
Number of crystals		12	10
Maximum dose for single data set	(e^−^/Å^2^)	1.26	0.63
For water standard	Gy	7.0 × 10^6^	3.5 × 10^6^
**Molecular replacement phasing**			
LLG^||^		2859	2292
TFZ^¶^		30.5	27.7
**Refinement**			
Resolution (Å)		131.3–3.20	131.3–3.20
		(3.29–3.20)	(3.29–3.20)
	*R*_work_	0.309	0.308
	*R*_free_	0.348	0.356
R.m.s. deviations			
	Bond lengths (Å)	0.004	0.004
	Bond angles (°)	0.731	0.725
Ramachandran plot (%)			
	Favored	91.7	92.4
	Allowed	7.85	7.14
	Outliers	0.45	0.50
All-atom clashscore		3.31	3.65

* *An averaged value for the merged datasets.*

† *Measured on the DE64 detector.*

‡ *Highest resolution shell is shown in parentheses.*

§ *R_merge_ = Σ_hkl_Σ_i_|I_hkl, i_ – < I_hkl_>|/Σ_hkl_Σ_i_ I_hkl, i_.*

|| *Log-likelihood gain (McCoy et al., 2007). LLG should be positive and high for the likely solution.*

¶ *Translation function Z score (McCoy et al., 2007). TFZ > 8 indicates the likely solution*.

**Table 2:** Data collection, crystallographic, and refinement statistics of Ph-BTBT-C10.

**Sample**		**Ph-BTBT-C10**
**Data Collection**		
Microscope		CRYO ARM 300
Wavelength (Å)		0.0197
Electron voltage (kV)		300
Energy filtration		-
Camera		DE64
Camera distance		
	IL1 (hexadecimal)	607E
	Nominal (mm)	500
	Measured (mm)	645
Temperature (K)		~293 (R.T.)
Rotation speed (°/s)		1.0
Rotation range		−68 to 68°
Rotation/frame (°/frame)		0.5
Dose/frame (e^−^/Å^2^/frame)^*^		0.005
Total datasets collected		33
**Crystallographic parameters**		
Space group		*P*2_1_/*a*
Cell dimensions		
	*a, b, c* (Å)	5.90, 7.51, 51.33
	*α, β, γ* (°)	90, 93.06, 90
Resolution (Å)		7.51–0.80
		(0.85–0.80)^†^
Completeness (%)		78.5 (80.0)
CC_1/2_ (%)		98.8 (88.6)
*R*_merge_^‡^		33.4 (93.3)
*I*/σ		4.2 (0.5)
Multiplicity		13.6 (13.7)
Solvent content (%)		1.0
Number of crystals		7
Maximum dose for single dataset	(e^−^/Å^2^)	1.3
For water standard	Gy	7.5 × 10^6^
**Direct phasing**		
CFOM, best^§^		112.5
CC, best^||^		61.1
**Refinement**		
Resolution (Å)		7.51–0.80
		(0.85–0.80)
	*R*_1_ (*F*_o_ > 4σ)	0.254
	*R*_1_ (all *F*_o_)	0.325

* *Measured on the DE64 detector.*

† *Highest resolution shell is shown in parentheses.*

‡ *R_merge_ = Σ_hkl_Σ_i_|I_hkl, i_ – < I_hkl_>|/Σ_hkl_Σ_i_ I_hkl, i_.*

§ *Combined figure of merit from the best trace. Correct solution has CFOM scores >80.*

|| *Correlation coefficient between the native intensities and those calculated from the best trace*.

Dose rate was estimated with the DE64 or K3 in counting mode before switching the microscope to diffraction mode. We found that counts measured by the K3 were fluctuated under this quite low-dose condition, and accepted counts by DE64 in this report. The setting of illumination was unchanged during data collection. The camera distance was calibrated from gold sputtered on carbon after the end of the data collection session.

### Data Processing and Structure Determination

Diffraction stacks, each of which comprises 276 raw frames, were first ×2 binned (2k × 2k pixels) for speeding up the following steps. Diffraction datasets were then subjected to automatic processing with KAMO (Yamashita et al., 2018), which carried out indexing, integration, scaling, and merging by using XDS (Kabsch, 2010a), DIALS (Winter et al., 2018), Pointless (Evans, 2011), XSCALE (Kabsch, 2010b), and BLEND (Foadi et al., 2013). The first and last exposed frames in each stack were excluded from this step. The camera distances were refined during the process (Kabsch, 2010a) from the initial values calibrated from the gold standard. The calibrated distance was adopted for Ph-BTBT-C10 due to a poorer convergence of the refinement.

The crystal structure of catalase was determined by molecular replacement starting from an atomic model of catalase by X-ray crystallography (PDB ID: 3NWL; Foroughi et al., 2011) using Phaser (McCoy et al., 2007) as described previously (Yonekura et al., 2015, 2019). The models were refined against the electron diffraction data using electron scattering factors with positional refinement of Phenix.refine (Afonine et al., 2012). Data and refinement statistics are shown in Table 1.

For Ph-BTBT-C10, possible lattice groups were examined for structure determination by the direct method using SHELXD (Sheldrick, 2010). A reasonable solution of the Ph-BTBT-C10 crystal structure was obtained with *P*2_1_/*a* with lattice parameters of *a* = 5.90 Å, *b* = 7.51 Å, *c* = 51.33 Å, α = γ = 90° and β = 93.06°.

The structure was then refined with SHELXL (Sheldrick, 2015) using anisotropic displacement parameters for all carbon and sulfur atoms. C–C and C–S bond distances were restrained based on the X-ray structure. Restraints in atomic displacement parameters (ADPs) were adjusted with SIMU, ISOR, and XNPD instructions so that ADPs were within reasonable values. The final *R*_1_ values are 0.254 (*F*_o_ > 4σ) and 0.325 (all *F*_o_). The high-resolution limit was determined to 0.80 Å according to *R*-factor values. Data and refinement statistics are summarized in Table 2.

**Figures 3A**, **4C,D**, **5C,D** were prepared with XQED (Yonekura et al., 2015), and **Figures 4A**, **5A,B** were prepared with Adxv (https://www.scripps.edu/tainer/arvai/adxv.html). **Figures 3B**, **4E** were prepared with PyMol (The PyMOL Molecular Graphics System, Schrödinger, LLC).

## Results and Discussion

### Data Collection With the DE64 Detector

**Figure 2:**
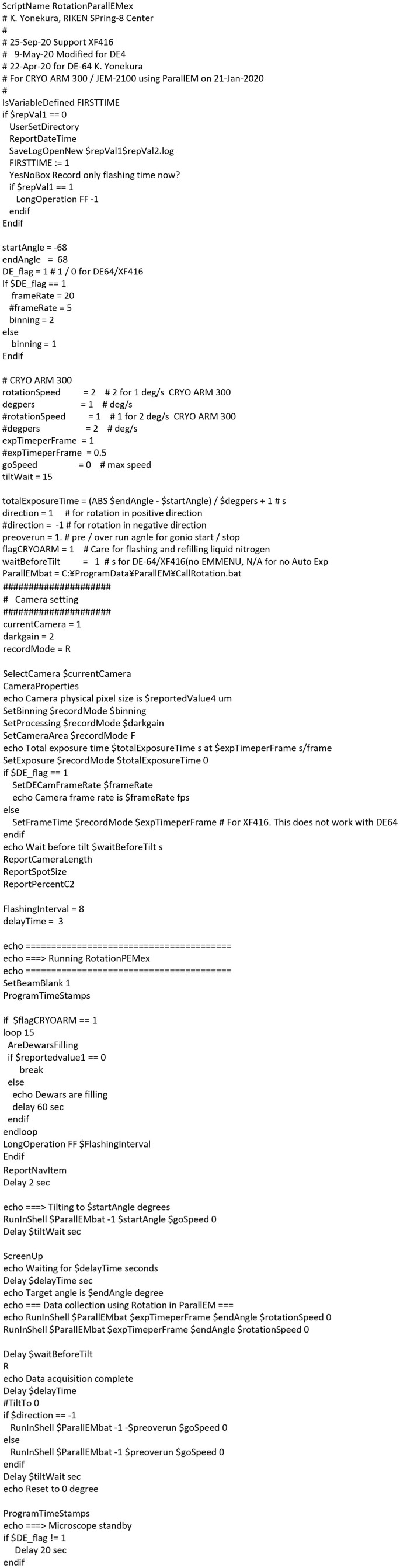
A SerialEM script for rotational data collection on the DE64. The script calls Rotation in ParallEM (Takaba et al., 2020) and can also be used for the XF416 without its camera control software EMMENU. The code is available as Supplementary Material 1.

We have analyzed two crystal structures of catalase and Ph-BTBT-C10. Rotational diffraction data from the crystals were collected on an active pixel sensor DE64 with a 300 kV electron beam. We always use the center beam stopper, and the sensor appeared to withstand the strong intensity around a direct beam but not be damaged. We previously developed and reported a semi-automated protocol for rotational data collection of electron diffraction patterns by combining SerialEM and ParallEM (Takaba et al., 2020). ParallEM calls standard camera control software of GATAN and TVIPS cameras through the GUI operation scripting language AutoIt (https://www.autoitscript.com/site/). However, no specific camera control software is provided for DE64, and SerialEM can be used, instead. Thus, we prepared a new SerialEM script, which does not use AutoIt, for this detector as in Figure 2.

We operated the detector at 20 fps in integration mode using the rolling shutter, summed 5 raw frames for catalase and 10 for Ph-BTBT-C10 as a movie stack and saved disk space thereby. By treating data in this way, there were no saturated pixels in the movie stacks with the illumination condition used for the data collection. We also tried data collection at 20 and 5 fps without summation of raw frames for Ph-BTBT-C10 crystals and did a slower rotation (1°/s) for catalase crystals, but found little effects on data statistics.

The detector also supports electron counting, but needs an extremely low dose rate. The recommend dose rate is between 0.005 and 0.025 e^−^/pixel/frame at the fastest frame rate (141 fps). Beyond this range, estimation of the deposited electron number on the sensor is inaccurate. Thus, it is impractical to use the counting mode for recoding electron diffraction patterns.

Thanks to task specialization among ParallEM and SerialEM (Takaba et al., 2020), the script and preparation before executing the script are simpler than those of a previous SerialEM script CRmov (de la Cruz et al., 2019), which is designed for the Thermo Fisher Scientific electron microscope. Average users takes <1 h for the microscope setup, whereas registration of x, y positions of crystals is still time-consuming and needs user labor. Once the registration is finished, the automated scheme can collect one rotational diffraction data set per 2~3 minutes without human supervision (Takaba et al., 2020).

### Catalase

**Figure 3:**
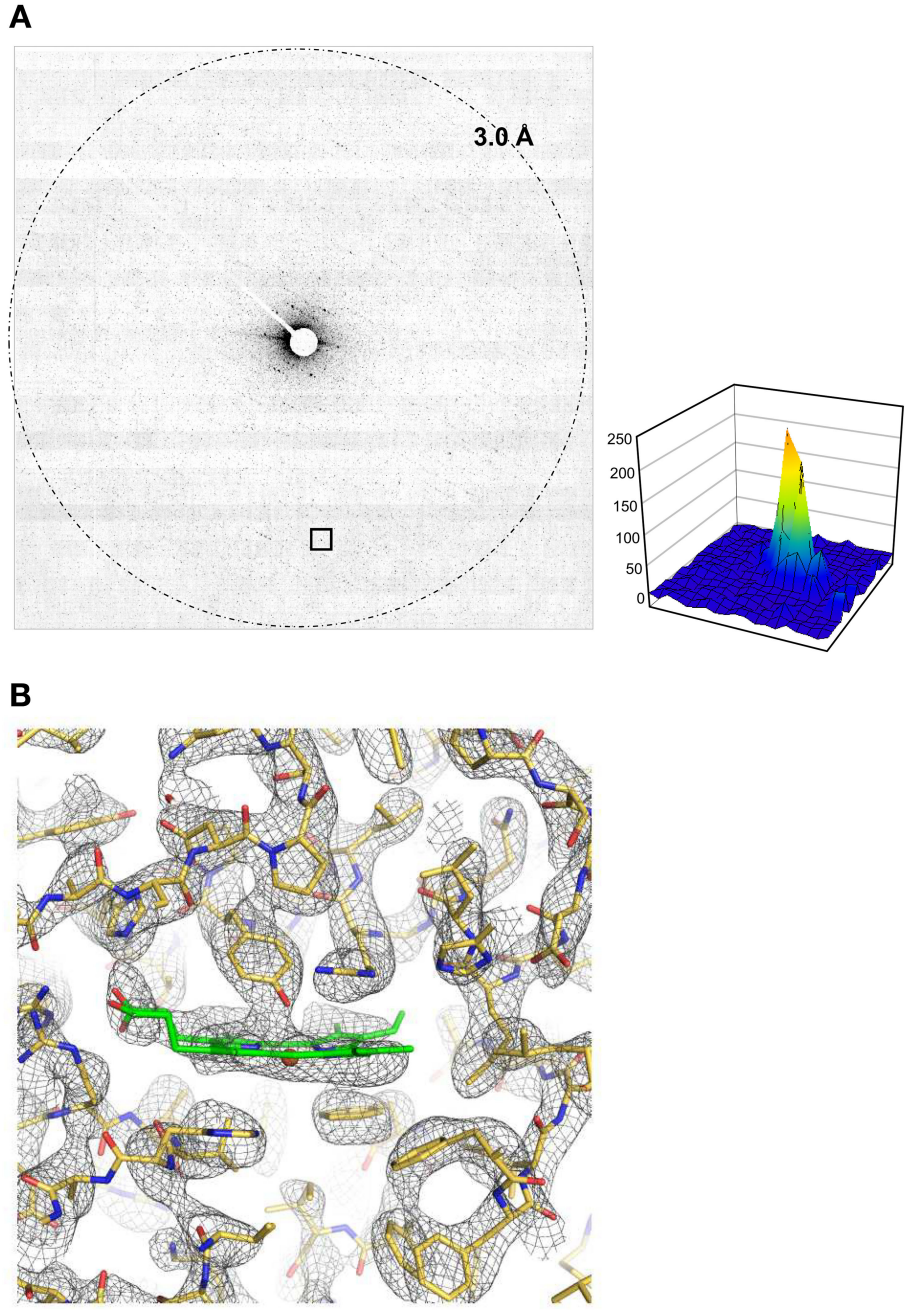
Structure analysis of catalase. **(A)** One frame covering a rotation of 0.5° at 0.005 e^−^/Å^2^ for 0.25 s exposure. An intensity profile in 19 × 19 pixels is shown on the right side for a diffraction spot at 4.3 Å resolution enclosed with a square at the bottom. **(B)** A Coulomb potential map (σ_A_-weighted 2 |*F*_obs_| – |*F*_calc_| maps) of catalase around the heme-binding site overlaid with the atomic model refined in this study. Calculated from the full rotational frames. The asymmetric unit of the crystal contains one tetramer of catalase, and non-crystallographic symmetry averaging was applied to the four molecules. Gray nets are contoured at 1.3σ, and carbon atoms in the model of heme is in green.

Plate-like crystals of catalase yield excellent electron diffraction patterns, but they are too thin for X-ray crystallography (Dorset and Parsons, 1975). Several crystal structures of catalase analyzed by electron 3D crystallography were reported so far (Nannenga et al., 2014b; Yonekura et al., 2015, 2019; Yonekura and Maki-Yonekura, 2016). Here we used a 1/6 lower dose rate for one frame and 1/5 ~ 1/4 total dose for 3D data collection compared with our previous datasets recorded on a scintillator-coupled detector GATAN OneView (Yonekura et al., 2019). Diffraction spots appear weak on one frame of a rotational dataset at this dose level (Figure 3A), while the DE64 showed low and flat background (Figure 3A) and noise from multiple readouts appears to be small. Total 53 diffraction datasets were processed, sorted, and merged in an automated manner (Yamashita et al., 2018). Despite weaker spots on one frame, most of the datasets was well-indexed, but the isomorphism among crystals was relatively poor as seen (Nannenga et al., 2014b; Yonekura et al., 2015). One resultant group consisting of 10 or 12 datasets with good quality and good isomorphism showed consistent lattice parameters (Table 1).

The crystal structure from these merged data was then phased by molecular replacement (McCoy et al., 2007) and refined in the standard way (Afonine et al., 2012). The crystal symmetry allows structure determination from the first half of the rotation frames, while the completeness is increased when using the full rotational frames (−68 to 68°; Table 1). Yet, these values are not excellent (Table 1) and the final resolution was slightly worse than those for the previous data (Yonekura et al., 2019) probably due to a low dose rate in data collection. Data statistics also strongly depends on the crystal quality of catalase (Yonekura et al., 2019) and the merging of multiple datasets may not improve the data quality much due to crystal isomorphism (Nannenga et al., 2014b; Yonekura et al., 2015). Nevertheless, the Coulomb potential map clearly resolves most of densities in the main and side chains and ligands (Figure 3B).

### Ph-BTBT-C10

Ph-BTBT-C10 is a representative material of organic semiconductors. It forms well-ordered thin layered crystals (Minemawari et al., 2014), whereas the crystallinity is not superb along the layers due to ~7–9 times longer repeat between the layers (*a* = 5.90 Å and *b* = 7.51 Å vs. *c* = 51.33 Å). This feature of this molecular species makes application of X-ray crystallography not straightforward, as crystallization of thick single crystals is often very difficult. Moreover, the thin-plane crystalline nature is important for functioning as semiconductor, and thicker crystals may not represent a true functional structure. Thus, we applied electron crystallography to Ph-BTBT-C10.

**Figure 4:**
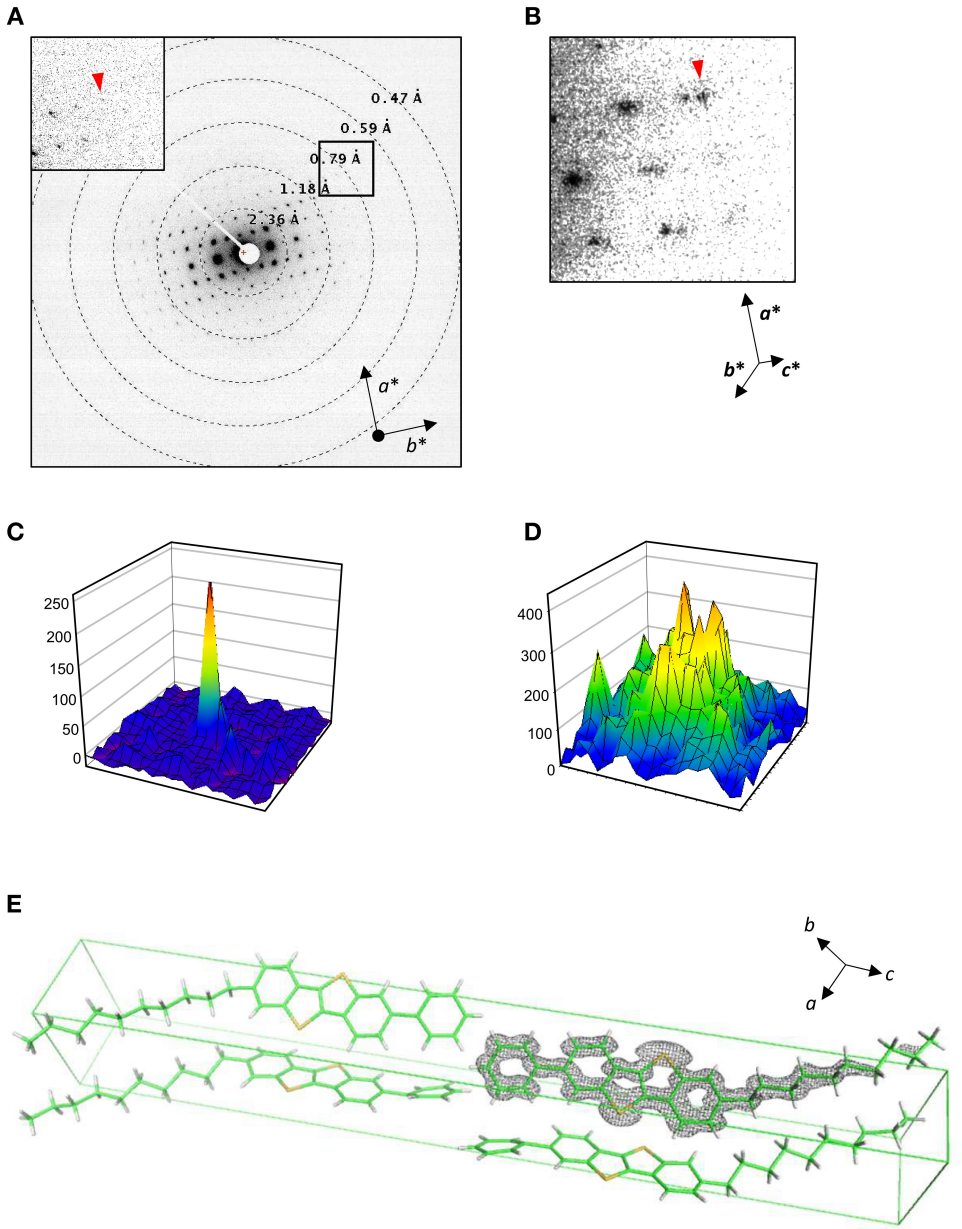
Structure analysis of Ph-BTBT-C10. **(A,B)** Electron diffraction patterns at lower tilt angles covering 4.5–5.0° **(A)** and a zoom-up at higher tilt angles covering −50 to −49.5° **(B)**. Inset in **(A)** shows a zoom-up of a square. Reciprocal cell axes are shown with arrows. **(C,D)** Intensity profiles in 19 × 19 pixels for diffraction spots with arrow heads in **(A)** and **(B)**, respectively. **(E)** Atomic models determined in this study, containing four molecules in the unit cell. A Coulomb potential map (σ_A_-weighted 2 |*F*_obs_| – |*F*_calc_| map) is overlaid on one molecule. Gray nets are contoured at 1.5σ. The cell axes are shown with arrows.

Rotational diffraction datasets were collected and processed in the same way as for catalase, except for the energy slit retracted. Energy filtration was less effective for these crystals containing no solvent molecules nor heavy atoms, and insertion of the energy slit limits the field of view with this shorter camera length (Maki-Yonekura et al., 2020). Each pattern yields clear diffraction spots up to ~0.7 Å in the a^*^-b^*^ plane (Figures 4A,C) but noisier along the c^*^ axis (Figures 4B,D). We found different layers were often stacked, and were able to carry out reliable indexing of diffraction spots for only eight datasets out of 33. The lattice parameters are similar to those of the previous X-ray structure (Minemawari et al., 2014), and the crystallographic symmetry was determined to be *P*2_1_/*a*, which is the same as in the X-ray structure (Minemawari et al., 2014).

Then, we solved the structure by the direct method from the merged data consisting of seven good datasets, and refined the structure. For the refinement, we tried both X-ray and electron scattering factors. The former yielded a better *R*_1_ value (0.254) than the latter did (0.300), whereas there were higher residual densities in the 3D map with X-ray scattering factors than that with electron factors. In contrast, we obtained better *R* values for other organic molecules with electron scattering factors. Current models for scaling of intensity and/or electron scattering factors used may not be suitable for some molecules in a given resolution range, due to dynamical scattering and scattering from charged atoms (Yonekura et al., 2015; Yonekura and Maki-Yonekura, 2016). We are now investigating this possibility and will report these issues elsewhere. Thus, this report shows the Ph-BTBT-C10 model refined with X-ray scattering factors.

The root mean square deviation for the X-ray structure is 0.20 Å. The Coulomb potential map is shown in Figure 4E. The BTBT part is particularly clear, although the map is elongated along the *c* axis and the alkyl tail is relatively poorly resolved most likely due to the missing wedge (Figure 4C). The tail part also shows higher temperature factors as in X-ray structure. Nevertheless, arrangement of molecules, which is key for these materials functioning as semiconductor, can be well-elucidated by electron 3D crystallography.

### Comparison With a Scintillator-Coupled Detector

**Figure 5:**
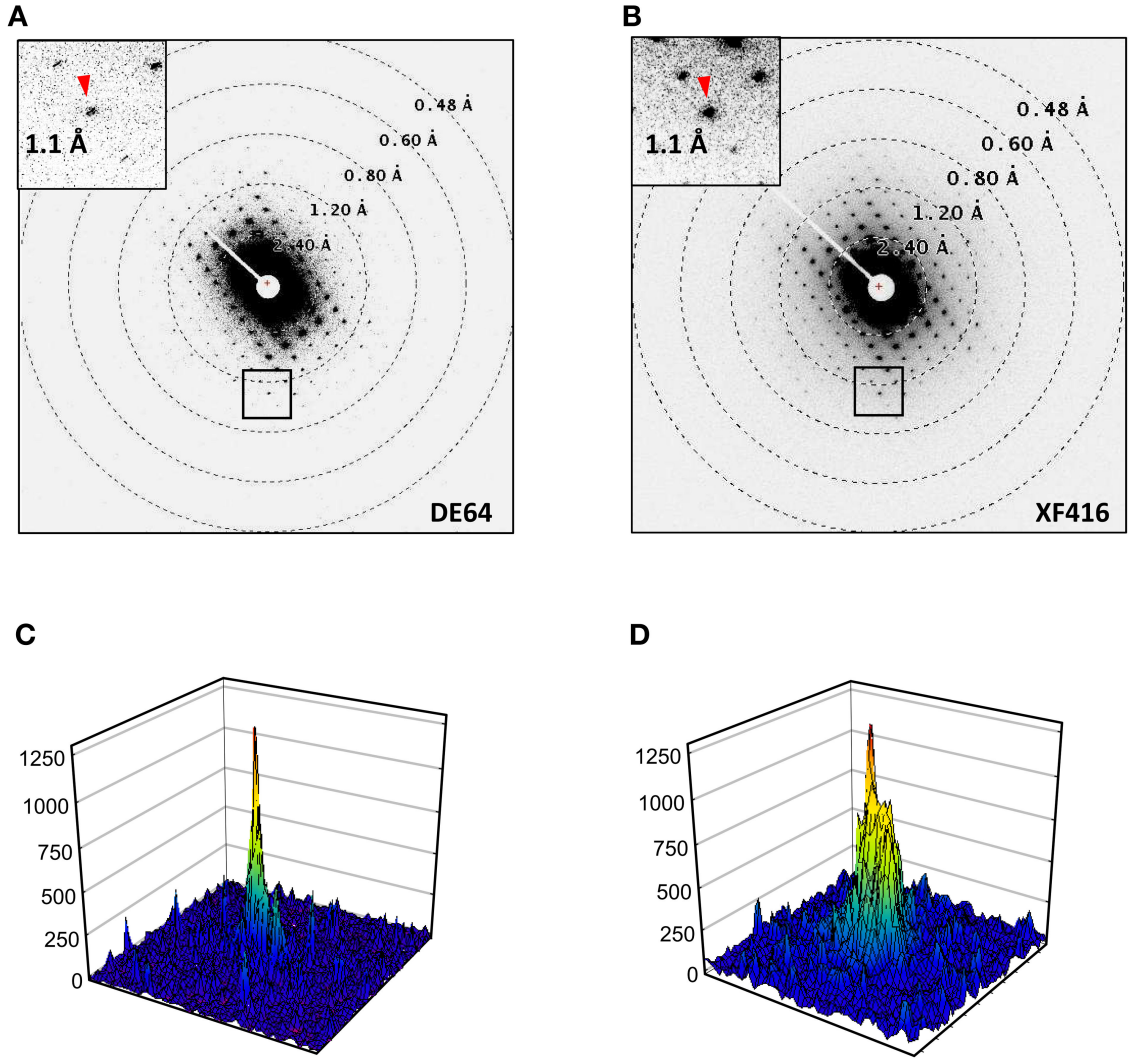
Electron diffraction patterns of a similar molecule to Ph-BTBT-C10. **(A)** Recorded on the DE64 with the same condition as in Figure 4 except for at 1s exposure/frame. **(B)** Reordered on the XF416 as in **(A)** except for a shorter camera length of 400 mm. **(C)** An intensity profiles (62 × 62 pixels) of a diffraction spot referred with an arrow head in **(A)**. **(D)** An intensity profile (52 × 52 pixels) of diffraction spot in **(B)**. The display areas in **(C)** and **(D)** were adjusted between the two cameras placed one above the other (Figure 1). Note that the peak profile is sharper and background noise is lower in **(C)** than those in **(D)**.

We compared electron diffraction patterns recorded on the DE64 (Figures 5A,C) and a scintillator-coupled CMOS camera XF416 (B and D). Crystals of a similar molecule to Ph-BTBT-C10 were used for this comparison. Again, the comparison is rather qualitative, as the data in Figures 5A,B were collected from different crystals. Nevertheless, the DE64 produced sharper diffraction spots and lower background noise than the XF416 did.

## Summary and Perspectives

The DE64 works well for electron 3D crystallography of protein and organic molecules at 300 kV as shown in the structure analyses above. Indeed, we have already succeeded in solving other new atomic structures including polypeptides and complex organic molecules with this system (manuscripts in preparation).

Radiation damage is serious in both X-ray analysis and cryo-EM, and previous studies observed that even a small amount of electron irradiation caused breaks of cysteine bonds (Hattne et al., 2018, 2019) and reduction of metal (Yonekura et al., 2015) in protein crystals. The radiation damage caused by single 300 kV electron is reduced by 49 and 20%, compared with 100 and 200 kV, respectively (Yonekura et al., 2019). Deposited energy with single 300 kV electron/Å^2^ was calculated to 5.6 × 10^6^ Gy (J/kg) for water (ICRU, 2014; Yonekura et al., 2019), where Gy is a standard unit in X-ray crystallography and related areas. Henderson limit, a criterion for a tolerable energy deposition on biological samples and widely used in X-ray crystallography, is ~2 × 10^7^ Gy (Henderson, 1990). The catalase structure here was obtained from a maximum exposure of 3.5 × 10^6^ M Gy for single dataset (Table 1), and this is 1/5.7 of Hendrson limit. Thus, our system would be suitable for electron 3D crystallography with less damaging, a smaller point spread, and less noise than using the scintillator coupled camera.

Still, detectors currently available are not perfect as mentioned, and an innovative detector technology may further advance electron 3D crystallography, as already seen in X-ray crystallography. In addition, there is a larger field-of-view cut with insertion of the energy slit for a camera distance shorter than 1000 mm (Maki-Yonekura et al., 2020). This could be improved in the future by the manufacturer and a new filter system be useful for accurate analysis of Coulomb potentials in molecules (Yonekura et al., 2015, 2018; Yonekura and Maki-Yonekura, 2016).

## Data Availability Statement

Atomic coordinates and structure factors for the crystal structures of catalase have been deposited in the Protein Data Bank under accession number 7DI8. A Crystallographic Information File (CIF) for Ph-BTBT-C10 is available as Supplementary Material 2 in this report.

## Author Contributions

KY, KT, SM-Y, and TH conceived the project. SI synthesized a sample. KT, SM-Y, and KY carried out data collection. KT analyzed the data. KT and KY wrote the manuscript. All of the authors joined the discussion of the results.

## Conflict of Interest

The authors declare that the research was conducted in the absence of any commercial or financial relationships that could be construed as a potential conflict of interest.

## Supplementary Material

Supplementary Material 1 A SerialEM script RotationParallEMex.txt.

Supplementary Material 2 A CIF file for Ph-BTBT-C10.
