# Corrigendum: Protein and Organic-Molecular Crystallography with 300kV Electrons on a Direct Electron Detector

**DOI:** 10.3389/fmolb.2021.749448

**Published:** 2021-08-18

**Authors:** Kiyofumi Takaba, Saori Maki-Yonekura, Satoru Inoue, Tatsuo Hasegawa, Koji Yonekura

**Affiliations:** ^1^ Biostructural Mechanism Laboratory, RIKEN SPring-8 Center, Sayo, Japan; ^2^ Department of Applied Physics, The University of Tokyo, Tokyo, Japan; ^3^ Advanced Electron Microscope Development Unit, RIKEN-JEOL Collaboration Center, RIKEN Baton Zone Program, Sayo, Japan; ^4^ Institute of Multidisciplinary Research for Advanced Materials, Tohoku University, Sendai, Japan

**Keywords:** DE64, energy filter, electron 3D crystallography (3D ED/MicroED), eEFD, CRYO ARM

In the original article, there was an error in the **Summary and Perspectives**. It was indicated that radiation damage caused by single 300 kV electron is reduced to 96 and 25%, compared with 100 and 200 kV, respectively. In fact, the correct values are by 49 and 20%. A correction has been made to the **Summary and Perspectives** section, paragraph 2.

“Radiation damage is serious in both X-ray analysis and cryo-EM, and previous studies observed that even a small amount of electron irradiation caused breaks of cysteine bonds (Hattne et al., 2018, 2019) and reduction of metal (Yonekura et al., 2015) in protein crystals. The radiation damage caused by single 300 kV electron is reduced by 49 and 20%, compared with 100 and 200 kV, respectively (Yonekura et al., 2019). Deposited energy with single 300 kV electron/Å^2^ was calculated to 5.6 × 10^6^ Gy (J/kg) for water (ICRU, 2014; Yonekura et al., 2019), where Gy is a standard unit in X-ray crystallography and related areas. Henderson limit, a criterion for a tolerable energy deposition on biological samples and widely used in X-ray crystallography, is ∼2 × 10^7^ Gy (Henderson, 1990). The catalase structure here was obtained from a maximum exposure of 3.5 × 10^6^ M Gy for single dataset (Table 1), and this is 1/5.7 of Hendrson limit. Thus, our system would be suitable for electron 3D crystallography with less damaging, a smaller point spread, and less noise than using the scintillator coupled camera.”

The authors apologize for this error and state that this does not change the scientific conclusions of the article in any way. The original article has been updated.

